# Blood-Brain Barrier, Blood-Brain Tumor Barrier, and Fluorescence-Guided Neurosurgical Oncology: Delivering Optical Labels to Brain Tumors

**DOI:** 10.3389/fonc.2020.00739

**Published:** 2020-06-05

**Authors:** Evgenii Belykh, Kurt V. Shaffer, Chaoqun Lin, Vadim A. Byvaltsev, Mark C. Preul, Lukui Chen

**Affiliations:** ^1^Department of Neurosurgery, Barrow Neurological Institute, St. Joseph's Hospital and Medical Center, Phoenix, AZ, United States; ^2^Department of Neurosurgery, School of Medicine, Southeast University, Nanjing, China; ^3^Department of Neurosurgery, Irkutsk State Medical University, Irkutsk, Russia; ^4^Department of Neurosurgery, Neuroscience Center, Cancer Center, Integrated Hospital of Traditional Chinese Medicine, Southern Medical University, Guangzhou, China

**Keywords:** fluorescence-guided surgery, blood-brain barrier, blood-tumor barrier, 5-aminolevulinic acid, fluorescein sodium, indocyanine green, enhanced permeability and retention, drug delivery

## Abstract

Recent advances in maximum safe glioma resection have included the introduction of a host of visualization techniques to complement intraoperative white-light imaging of tumors. However, barriers to the effective use of these techniques within the central nervous system remain. In the healthy brain, the blood-brain barrier ensures the stability of the sensitive internal environment of the brain by protecting the active functions of the central nervous system and preventing the invasion of microorganisms and toxins. Brain tumors, however, often cause degradation and dysfunction of this barrier, resulting in a heterogeneous increase in vascular permeability throughout the tumor mass and outside it. Thus, the characteristics of both the blood-brain and blood-brain tumor barriers hinder the vascular delivery of a variety of therapeutic substances to brain tumors. Recent developments in fluorescent visualization of brain tumors offer improvements in the extent of maximal safe resection, but many of these fluorescent agents must reach the tumor via the vasculature. As a result, these fluorescence-guided resection techniques are often limited by the extent of vascular permeability in tumor regions and by the failure to stain the full volume of tumor tissue. In this review, we describe the structure and function of both the blood-brain and blood-brain tumor barriers in the context of the current state of fluorescence-guided imaging of brain tumors. We discuss features of currently used techniques for fluorescence-guided brain tumor resection, with an emphasis on their interactions with the blood-brain and blood-tumor barriers. Finally, we discuss a selection of novel preclinical techniques that have the potential to enhance the delivery of therapeutics to brain tumors in spite of the barrier properties of the brain.

## Introduction

Gliomas account for nearly 80% of primary malignant tumors in the central nervous system (CNS) (1). Current recommended treatment for glioblastoma includes maximal safe resection, radiotherapy, temozolomide, and alternating electric field therapy (2). Despite all treatment efforts, glioma recurrence is inevitable due to the invasive nature of the tumor hampering complete tumor resection (3). Although it is practically impossible to eradicate every glioma cell surgically, increasing the precision of glioma removal with more accurate margin delineation predicts better treatment outcomes and preservation of quality of life (4–7). Because of the infiltrative growth pattern of gliomas, the tumor boundary is a mixture of tumor cells, reactive glial and immune cells, as well as normal brain cells. Such architecture complicates delivery of drugs to the invasive border region and restricts complete tumor resection. Margin delineation during resection is difficult and is inherently biased by both the subjective experience of the surgeon and the technical limitations of the operating microscope. Because of these factors, the goal of maximal safe resection of gliomas remains difficult to achieve, if not elusive.

In recent years, a number of intraoperative optical techniques, including fluorescence-guided surgery, have been developed to improve intraoperative visualization of cancers. These techniques can specifically label tumor cells, helping neurosurgeons to more objectively determine the boundary between the abnormal and surrounding normal tissues and to achieve more informed, maximum, and safe tumor resection. The impact of fluorescence techniques on extending progression-free and overall survival in high-grade gliomas is yet to be fully realized (8, 9). The most common drugs used for fluorescence-guided resection of high-grade gliomas are 5-aminolevulinic acid (5-ALA), fluorescein sodium (FLS), and indocyanine green (ICG), while multiple other fluorescent probes are currently in Phase I-II stages of investigation. Despite their mitigated success in clinical application for high-grade gliomas, current fluorescent labels often do not stain the overall area of the tumor, especially near the tumor margin, and do not delineate tumor margins in low-grade brain tumors. These difficulties are likely due to the effects of the blood-brain barrier (BBB), which maintains the sensitive environment of the brain by preventing the passage of blood-borne proteins, drugs, inflammatory cells, and other solutes. Consequently, delivery of fluorescent markers into the brain is also prevented, limiting the effectiveness of optical imaging techniques. Breakdown of the BBB is common in high-grade gliomas and brain metastases, producing what is known as the blood-brain tumor barrier (BBTB). It is this dysfunctional barrier that enables the extravasation of fluorescent markers into tumor tissue. However, the BBTB is more competent in low-grade gliomas and at the invasive border of high-grade gliomas (10, 11), complicating tumor staining (12, 13). Clearly, it would be advantageous to understand and manipulate the BBB and BBTB to overcome regional barriers to drug delivery and to target tumor cells where the BBTB is less disrupted.

In this review, we summarize the structure and function of the BBB and BBTB and their interactions with fluorescence visualization techniques for the optical guidance of invasive brain tumor resection. We review novel techniques that show potential for trans-BBB delivery of labels and therapeutics to elucidate the extent of brain tumors and to guide brain tumor surgery.

## The Blood-Brain Barrier

**Figure 1:**
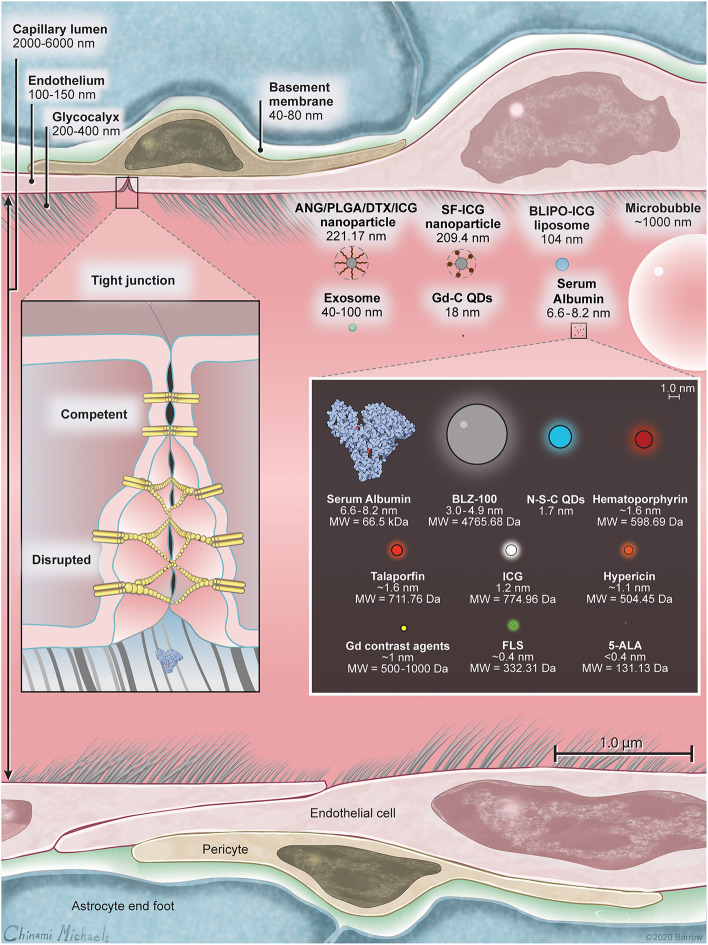
A scale illustration of blood-brain barrier (BBB) structure with a selection of drugs and fluorescent markers discussed in this paper. Left inset shows the structure of the BBB including luminal glycocalyx, tight junctions (TJs), and the membranes of the neighboring endotheliocytes. In intact BBB, TJs secure the paracellular transport, while in disrupted BBB or in BBTB, TJs are deficient and allow for paracellular transport of large molecules through the interendothelial slits and pores. Human serum albumin is included because many small-molecule drugs (including ICG and FLS) bind extensively to serum proteins upon intravascular administration, thus changing their ability to cross the BBB. Right inset is scaled relative to the molecules within. Listed in nanometers are hydrodynamic diameters of molecules, when available, or molecular chain length estimated using PyMOL (The PyMOL Molecular Graphics System, Version 2.3 Schrödinger, LLC). 5-ALA, 5-aminolevulinic acid; ANG/PLGA/DTX/ICG, angiopep2/poly(lactic-co-glycolic acid)/docetaxel/indocyanine green; BLIPO-ICG, biomimetic liposome conjugated to indocyanine green; FLS, fluorescein sodium; Gd, gadolinium; Gd-C QD; gadolinium-carbon quantum dot; ICG, indocyanine green; N-S-C QD, nitrogen and sulfur co-doped carbon quantum dot; SF-ICG, silk fibroin indocyanine green conjugated. *Used with permission from Barrow Neurological Institute, Phoenix, Arizona*.

The BBB is primarily composed of a continuous layer of non-fenestrated capillary endothelial cells covered by the glycocalyx and securely connected by a net of intercellular tight junctions (TJs) and adherens junctions, a basement membrane, pericytes, and perivascular astrocyte end-foot processes. Conceptually, the BBB contains three barriers arranged sequentially from the blood to the brain: the glycocalyx, endothelium, and extravascular compartment (14). A scaled schematic of these barriers is shown in Figure 1. Knowledge of the structure and function of these barriers is important not only for understanding the pharmacology of optical tracers in brain tumors and surrounding normal brain, but also for informing the design of novel drugs that can efficiently target invading tumor cells hidden behind regions of relatively intact BBTB.

### Glycocalyx

The glycocalyx is an ~300-nm thick (15), gel-like structure on the luminal membrane of the endothelium consisting of negatively charged proteoglycans, glycosaminoglycans, and glycoproteins anchored in the luminal membrane by transmembrane proteins (16). A functional glycocalyx prevents the adhesion of circulating cells to the endothelium and serves as an initial malleable, sieve-like barrier to large molecules. For example, the concentration of intravascularly administered 150-kDa dextrans decreases by almost 50% within the glycocalyx layer, while the concentration of FLS (376 Da) remains above 90%, when compared to the center of the vessel (14, 17).

### Endothelium

Endothelial cells create an ~200-nm thick, highly functionalized wall with luminal and abluminal membranes. These cells are tightly interconnected by TJs, lack fenestrations, and have a diminished number of pinocytic vesicles, limiting the transport of solutes across the BBB (18). Given these barrier properties, there are two main ways molecules pass the endothelial layer: via paracellular diffusion or using transcellular mechanisms.

**Figure 2:**
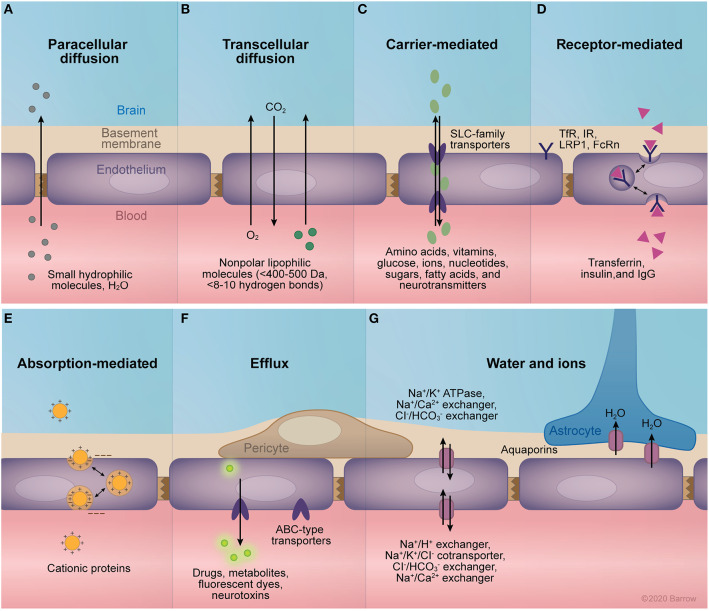
Transport mechanisms of the blood-brain barrier (BBB). **(A)** Paracellular diffusion through intact tight junctions is possible for small, hydrophilic molecules and water. **(B)** Transcellular diffusion of small non-polar lipophilic molecules. **(C)** The facilitated diffusion of small molecules across the endothelium is mediated primarily by members of the solute carrier family of transporters, which transport a range of molecules including amino acids, vitamins, glucose, ions, nucleotides, sugars, fatty acids, and neurotransmitters. **(D)** Receptor-mediated transport allows for the transcellular passage of larger molecules across the endothelium via their binding to specific receptors. These receptors trigger endocytosis by conformational changes in cytoplasmic domains, allowing for the transcytosis or endocytosis of bound molecules. Examples of receptors expressed at the BBB include the transferrin receptor, insulin receptor, low-density lipoprotein related receptor 1, and the neonatal Fc receptor. **(E)** Cationic proteins can traverse the BBB via binding to negatively charged cell-surface molecules expressed in the capillary lumen, allowing for their transcytosis across the endothelium. **(F)** Members of the adenosine triphosphate (ATP) binding cassette family of transporters are expressed at the central nervous system endothelium, mediating the active efflux of a large selection of drugs, metabolites, neurotoxins, and fluorescent markers. **(G)** Proper osmolarity of the endothelial basement membrane and the adjacent brain interstitium is mediated by the selective expression of ion pumps and channels. Namely, the Na^+^/K^+^ ATPase, Na^+^/Ca^2+^ exchanger, and Cl^−^/${\text{HCO}}_{3}^{-}$ exchanger regulate abluminal ion equilibrium, while the Na^+^/H^+^ exchanger, Na^+^/K^+^/Cl^−^ cotransporter, Cl^−^/${\text{HCO}}_{3}^{-}$ exchanger, and Na^+^/Ca^2+^ control ionic equilibrium with the blood. Additionally, expression of aquaporins 1 and 4 mediate the passive flow of water across endothelial cell and astrocyte membranes, respectively. ABC, adenosine triphosphate–binding cassette; FcRn, neonatal Fc receptor; IgG, immunoglobulin G; IR, insulin receptor; LRP1, low-density lipoprotein related receptor; SLC, solute carrier; TfR, transferrin receptor. *Used with permission from Barrow Neurological Institute, Phoenix, Arizona*.

Paracellular diffusion is significantly limited by the fence created by the continuous network of TJ complexes. TJs seal interendothelial clefts and anchor to the cytoskeleton, providing structural support. Brain endothelium differs from peripheral endothelium and epithelium in its expression of TJ proteins (high expression of occludin and claudin-5), which creates a tighter TJ network (19). Furthermore, alteration in the expression of the transmembrane proteins composing the TJs and their interactions modulate the gating of paracellular diffusion, allowing for dynamic control of paracellular diffusion (Figure 2A).

Transcellular diffusion is limited to those molecules that are relatively small, uncharged, and lipophilic (Figure 2B). More specifically, the intact BBB allows diffusion of hydrophobic molecules that are less than 400–500 Da in size and form fewer than 8 to 10 hydrogen bonds with water (20). For ideal delivery, a drug should have an octanol:water partition coefficient (P_ow_) of between 10:1 and 100:1 (21) or a total polar surface area less than 90 Å^2^ (22). Coupled with endothelial TJs, this barrier to diffusion allows the endothelial expression and modulation of select transporters and receptors to dictate the flow of large molecules across the BBB (Figures 2C–G). Transporters at the endothelial cell membrane are primarily members of the solute carrier (SLC) family of passive membrane transporters (23–25) or of the adenosine triphosphate-binding cassette (ABC) family of active transporters (26–28). In contrast, the receptors expressed at the BBB are diverse, including the transferrin receptor (29), neonatal Fc receptor (30, 31), and low-density lipoprotein receptor 1 (LRP1) (25).

### Extravascular Compartment

Other components of the BBB are pericytes, which cover 22–32% of the surface of the capillaries (32), the foot processes of neighboring astrocytes that completely encase the brain microvasculature (33), microglia, and neurons. These surrounding cells secrete signals that contribute to BBB development, provide physical and metabolic support, and are necessary to maintain dynamic integrity of the BBB (34–40). Altogether, these elements form a so-called neurovascular unit, capable of responding dynamically to local changes in physiology and environment (38, 41, 42). The composition, development, and regulation of the BBB have been previously reviewed in greater detail (21, 25, 38, 41–44), including a recent review of BBTB (45).

## The Blood-Brain Tumor Barrier

### The Role of Vascular Endothelial Growth Factor in the BBTB

A major driver of BBB compromise, especially in high-grade gliomas, is tumor-secreted vascular endothelial growth factor (VEGF). The increased metabolic rate of high-grade gliomas results in local hypoxia and upregulation of hypoxia inducible factor-1, which stimulates the production of VEGF. Secreted VEGF then induces breakdown of existing BBB architecture and growth of structurally altered capillaries from the existing vessels (46, 47). This induced tumoral vascular endothelium displays an abnormal expression profile of transporters and receptors in order to accommodate the high metabolic demands of associated tumor cells (48–52). Unlike the normal brain vessels from which they originate, newly formed capillaries are structurally altered and are more permeable than even non-BBB peripheral capillaries (53). Although the abnormal microvasculature of high-grade brain neoplasms is different from that of non-brain solid tumors, both are similarly hyper-permeable when compared with normal capillaries.

### Degree of BBTB Endothelium Permeability

The large size of interendothelial clefts and transendothelial fenestrations as well as their increased number contribute to the high degree of BBTB permeability. The degree of BBB and BBTB permeability is traditionally measured by using different-sized fluorescently labeled dextrans (54–58). The customizability of uniformly sized dextrans and their reactivity with fluorescent molecules such as fluorescein isothiocyanate (FITC) create easily assembled probes that can be visualized for interrogating BBB permeability. The average transendothelial pore size in intracranially implanted tumors is significantly smaller than that of extracranially implanted tumors (210–550 nm compared to 380–2,000 nm) (53). However, the hyperpermeability of the tumor capillaries to fluorescently labeled albumin (≈7 nm, 66 kDa) was associated with the number of pores, rather than their size (53). In average-size tumor vessels, about 30% had fenestrations and about 10% had open junctions (53). In a series of experiments with nine various-sized nanoparticles that tried to identify the maximal fenestration size in RG-2 glioma BBTB, 597 kDa molecules did not transgress the BBTB, while 330 kDa and smaller nanoparticles passed the BBTB (59).

To better portray the degree of BBTB hyperpermeability, tumor endothelium could be compared to non-brain endothelium, which is categorized into three groups based on permeability. (1) *Continuous non-fenestrated endothelium* is found in skin, heart, and lungs and is usually impermeable to molecules larger than 4–6 nm in diameter, but it may have intermittent discontinuities in TJs that create intercellular slits of up to 20 nm (60). (2) F*enestrated endothelium* of the intestines, kidneys, and choroid plexus has transcellular pores 25–60 nm in diameter, which are sealed by 5–6 nm thick diaphragms (61). (3) *Discontinuous endothelium* of the liver, spleen and bone marrow has large, 100–200 nm fenestrations without an underlying basement membrane (61). Consideration of the permeability of the various capillary types is important for the development of drugs, especially those that rely on the enhanced permeability and retention (EPR) effect for delivery.

### Heterogeneity of the BBTB

Tumor-mediated changes in the BBB may vary with tumor type, volume, stage, and anatomical location, or even within the same tumor (62). The severity of barrier compromise ranges significantly, from critical disruption comparable to the vasculature in solid, non-brain neoplasms to mild compromise found in neurodegenerative disease, stroke, diabetes, and obesity, among other pathologies (18, 21, 43). Indeed, studies have shown that brain tumors possess all three distinct types of endothelium: non-fenestrated continuous, similar to normal cerebral blood vessels; continuous fenestrated; and discontinuous endothelium (63–65).

**Figure 3:**
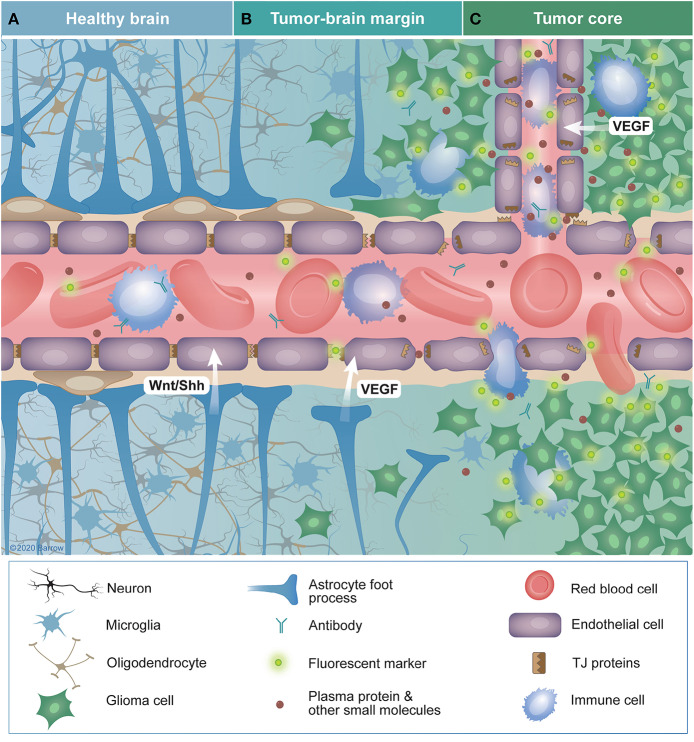
Characteristics of the blood-brain (BBB) and blood-brain tumor barriers (BBTB) in glioma. **(A)** The healthy BBB selectively impedes the diffusion of blood contents across the central nervous system (CNS) endothelium. Controlled expression of tight-junction proteins and the endothelial cells themselves provide a physical barrier to the passage of these solutes into the brain parenchyma. Additionally, intimate associations among endothelial cells, pericytes, astrocytes, and neurons (the neurovascular unit) promote the continued integrity of the BBB. Sonic hedgehog and Wnt-family proteins secreted by astrocytes and pericytes are crucial for BBB maintenance. **(B)** In tumor margin zones, glioma cells may infiltrate into otherwise healthy parenchyma, causing disruption of local neural structures, including the BBB. This infiltration disrupts the connections between members of the neuro vascular unit, leading to the downregulation of tight-junction proteins and degradation of the BBB through tumor-secreted vascular endothelial growth factor. The resulting vascular permeability leads to the extravasation of blood contents, including solutes, antibodies, fluorescent markers, and immune cells. Alternatively, glioma cells may infiltrate in such a way that they do not disrupt the BBB, effectively shielding themselves with an intact BBB. **(C)** Within the tumor core, BBB disruption is often the greatest, especially in high-grade gliomas. The increased density of highly metabolically active cells in this area promotes hypoxia-driven expression of VEGF that promotes angiogenesis. These new vessels are often immature, lacking typical CNS barrier properties, leading to characteristics such as aberrant transporter expression, increased interstitial pressure, and edema. TJ, tight junctions; VEGF, vascular endothelial growth factor; Wnt/Shh, Wnt and sonic hedgehog family proteins. *Used with permission from Barrow Neurological Institute, Phoenix, Arizona*.

In low-grade gliomas, the structure and function of the BBTB largely resembles that of the normal BBB (66). In grade III gliomas, the microvasculature surface area and vascular diameter are higher compared with low-grade gliomas (67). In high-grade gliomas, the BBB is significantly altered, leading to associated edema (68) and gadolinium-based contrast accumulation. However, even in high-grade gliomas there are regions with vascular density and integrity within the same range as that of normal cerebral white matter, especially in non-enhancing regions or necrotic, avascular regions with decreased perfusion (10, 69, 70). Interestingly, this disruption does not necessarily correlate with the degree of infiltration of normal brain by tumor cells. A single infiltrating glioblastoma cell can cause the foot processes of astrocytes to migrate away from vascular endothelial cells, leading to the formation of localized fractures in the BBB (71). Alternatively, infiltrating glioma cells may be shielded by an intact BBB from intravascularly administered diagnostic and therapeutic agents (72, 73). This spectrum of BBB disruption seen in glioma is shown in Figure 3. An additional layer of complexity is added by the regional heterogeneity of immune cell populations that influence the BBTB in gliomas (74–76).

Glucocorticoids that are frequently used to manage peritumoral edema also influence the BBTB, reducing transendothelial flow by shrinking interendothelial gaps and increasing formation of intercellular junctions (77). However, preoperative steroid use was not associated with the efficacy of FLS staining of high-grade glioma tissue for wide-field fluorescence guidance and thus is unlikely to affect delivery of optical agents to the core of the tumor (78).

These characteristics depict the BBTB as a heterogeneous combination of preexisting and newly formed blood vessels, which provide nutrients and oxygen to the tumor. Although the BBTB is disrupted in the tumor core, it may retain characteristics of an intact BBB in certain areas, thus creating a barrier that, while compromised, still hinders delivery of diagnostic agents to the tumor, diminishing the diagnostic accuracy of intraoperative optical guidance techniques (52, 79). The fact that the BBTB is so disrupted in the core of high-grade gliomas leads some scientists to question the BBB as the major factor that limits the effectiveness of chemotherapy in these tumors (11). Therefore, in order to improve these techniques for brain tumor surgery, we first need to understand the mechanisms that govern delivery of various optical labels across the heterogeneous BBTB in various tumor types. We also discuss approaches to circumvent the intact portions of the BBTB for more effective delivery of optical agents.

## Current Strategies for Delivery of Tumor Markers Across the BBB and BBTB

Efficacious drug delivery to the CNS has been difficult. The disrupted nature of the BBTB in high-grade gliomas permits increased delivery of drugs to the tumor core compared to surrounding normal brain, where the BBB is generally intact. Still, the invasive nature of high-grade gliomas and other non-enhancing brain tumors (80), in which cells migrate beyond the visible borders of gadolinium enhancement (81), presents a challenge for accurate delineation of the extent of tumor growth, for accurate diagnosis, and for intraoperative guidance.

Although many methods have been explored for drug delivery across the heterogeneous BBB and BBTB, these barriers remain a major obstacle (20, 21, 24, 52, 64, 82–92). In general, such methods can be classified into five broad categories: (1) passive delivery, (2) EPR of the BBTB, (3) BBTB disruption, (4) BBTB bypass, (5) BBTB targeting. We briefly discuss these categories of drug delivery and relate them to the current clinical tools and novel advancements for intraoperative optical guidance in neuro-oncology. Tumor markers reviewed in this manuscript are summarized in the Table 1.

**Table 1:** Preclinical and early clinical agents for delivery of optical labels to brain tumors discussed in this review.

**Agent**	**Interaction with BBB/BBTB**	**Stage of development**	**References**
**UNTARGETED AGENTS**			
[^18^F]-1; an [^18^F]-labeled dasatinib derivative for ^18^F-PET and fluorescence visualization *in vivo*	None; investigated using CED to bypass the BBB in mouse models	Preclinical	(93)
Collagen-based, ^131^Cs-laden wafer for intracranial brachytherapy	None; intracranial implant to bypass the BBB	Clinical	(94)
PEGylated FLS	None; improved passive accumulation within tumor tissues via the EPR effect in U251 orthotopic mouse glioma	Preclinical	(95)
Albumin-bound 5-aminoFLS	None; improved passive accumulation within tumor tissues via the EPR effect intraoperatively	Clinical	(96, 97)
Second-window ICG	None; passive accumulation within tumor tissues via a delayed EPR effect	Clinical	(98–102)
Aptamer-conjugated PEGylated quantum dots	None; passive accumulation via the EPR effect and targeting via EGFRvIII-targeted aptamers in orthotopic mouse gliomas	Preclinical	(103)
Silk fibroin-ICG nanoparticles	None; improved passive accumulation within orthotopic C6 mouse glioma cells via the EPR effect	Preclinical	(104)
**EPR Enhancers**			
PGI2 agonists	Dilation of tumor vasculature to increase extravasation and accumulation via the EPR effect	Preclinical	(105)
TNF-a		Preclinical	(106)
Intraarterial angiotensin II		Clinical	(107)
**ANTIBODY/AFFIBODY-BASED AGENTS**			
ABY-029, an anti-EGFR affibody conjugated to the near infrared probe IRDye 800CW	Targeting to EGFR receptors overexpressed on glioma cells	Clinical	(108), NCT02901925
FITC-conjugated anti-EGFR antibody		Preclinical	(109)
IRDye 800CW-conjugated anti-EGFR antibody		Preclinical	(110)
**OTHER TARGETED AGENTS**			
ANG2, a synthetic peptide shown to target the BBB	RMT via low-density lipoprotein receptor 1 targets both BBB and BBTB	Clinical	(111, 112), NCT01967810, NCT02048059
ANG2/PLGA/DTX/ICG probe, an ANG2-coated, ICG- and docetaxel-laden PLGA nanoparticle	RMT via low-density lipoprotein receptor 1 targets both BBB and BBTB	Preclinical	(113)
ANG2/PEG-UCNP, an ANG2-conjugated, gadolinium-laden fluorescent upconversion nanoparticle	RMT via low-density lipoprotein receptor 1 targets both BBB and BBTB	Preclinical	(114)
AsT, a TGN- and AS1411-conjugated Cy3 fluorescent probe	Endothelial cell uptake and transcytosis into the brain via TGN-conjugation by an unknown mechanism and AS1411 aptamer-mediated glioma cell targeting	Preclinical	(115)
Lysine-methotrexate prodrug	CMT via L-type amino acid transporter 1 targets the BBB in a mouse model	Preclinical	(116)
Aspartate-, 2-amino-apidic acid-, and phenylalanine-conjugated dopamine prodrugs	CMT via large neutral amino acid transporter 1 targets BBB	Preclinical	(117)
ApoE3-labeled porphyrin-lipid nanoparticles	RMT via low-density lipoprotein receptor 1 targets both BBB and BBTB in a mouse model	Preclinical	(118)
Transferrin-functionalized lipid nanoparticles laden with temozolomide and bromodomain inhibitor JQ1	RMT via transferrin receptor in a mouse model	Preclinical	(119)
BLZ-100 (tozuleristide), ICG-conjugated chlorotoxin	RMT via binding to chloride channels to trigger endocytosis at the BBB or BBTB and MMP-2 binding for targeting of glioma cells	Clinical	(120–123), NCT03579602
IRDye 800CW-cyclic-RGD peptide	Targeting to integrin receptor overexpression on tumor vasculature and on tumor cells in a mouse glioma model	Preclinical	(124)
BLIPO-ICG, ICG-laden biomimetic proteolipid liposomes	Passive accumulation via the EPR effect and targeting to glioma cells via functionalization with glioma-derived surface markers in a mouse glioma model	Preclinical	(125)
**5-ALA-COUPLED AGENTS**			
5-ALA + MEK inhibitor selumetinib	Inhibition of ABCB1-mediated PpIX efflux and ferrochelatase-mediated PpIX metabolism at the BBB or BBTB in a mouse model	Preclinical	(126)
**5-ALA** **+** **efflux pump inhibitors**			
Ko143	Inhibition of ABCG2-mediated PpIX efflux at the BBB or BBTB	Preclinical	(127, 128)
Gefitinib		Preclinical	(129)
Imatinib mesylate		Preclinical	(130)
5-ALA + iron chelators	None; inhibition of PpIX metabolism to heme via ferrochelatase in tumor cells	Preclinical	(131–134)
**5-ALA** **+** **differentiation agents**			
Calcitriol	None; enhanced PpIX accumulation via upregulation of coproporphyrinogen oxidase in epithelial cancer and glioma cell cultures	Preclinical	(135, 136)
Vitamin E	None; enhanced PpIX accumulation via upregulation of coproporphyrinogen oxidase in epithelial cancer cell culture	Preclinical	(137)
5-ALA + heme oxygenase inhibitors	None; accumulation of PpIX via inhibition of downstream metabolism of heme by heme oxidase	Preclinical	(138, 139)

*^18^F, Fluorine-18; ^131^Cs, Cesium-131; 5-ALA, 5-aminolevulinic acid; ABCB1, ATP-binding cassette subfamily B member 1; ABCG2, ATP-binding cassette subfamily G member 2; ANG2, angiopep-2; ApoE3; apolipoprotein E3; BBB, blood-brain barrier; BBTB, blood-brain tumor barrier; CMT, carrier-mediated transport; DCT, docetaxel; EPR, enhanced permeability and retention; FLS, fluorescein sodium; ICG, indocyanine green; MEK, mitogen-activated protein kinase kinase; MMP-2, matrix metalloproteinase 2; PEG, polyethylene glycol; PET, proton emission tomography; PLGA, poly(lactic-co-glycolic acid); PpIX, protoporphyrin IX; RMT, receptor-mediated transport; UCNP, up-conversion nanoparticle*.

### Passive Delivery

Passive delivery through the intact BBTB is challenging, as most small lipophilic drugs that could diffuse through the BBB are not targeted and exhibit toxicity in high doses, and most hydrophilic molecules do not pass through the narrow TJs of the BBB (140, 141).

Passive delivery of small molecules (<40 kDa) through the disrupted BBTB proceeds down a blood-tumor diffusion gradient, similarly to drug delivery to non-brain tumors. Interestingly, low-molecular-weight drugs usually have a lower “tumor-to-normal brain” distribution ratio compared with larger molecules. This difference is due to the rapid wash out of the small-molecule drugs from the extracellular space and clearance from the blood (142). Accumulation of small molecular labels in tumor tissues peaks within minutes and gradually decreases within 4–6 h. Fluorescence-guided surgery within this time window of first-pass accumulation and clearance is still feasible, mainly because a healthy BBB limits the extravasation of these labels (for example FLS, ICG, and others) to areas of tumoral BBB degradation.

### Enhanced Permeability and Retention

The EPR mechanism was initially described for non-brain solid tumors in 1986 (142, 143). This mechanism is based on four main components: (1) hypervascularization of the tumor, (2) enhanced permeability of tumor vasculature, (3) hampered absorption of macromolecules back into the vasculature, and (4) reduced drainage of molecules through the lymphatic system. Because the BBTB in high-grade gliomas is even more permeable than fenestrated non-brain capillaries, many optical guidance drugs reach the tumor via this mechanism. Furthermore, the lack of a lymphatic system in tumors prevents the clearance of large molecules and lipids from the interstitial space, greatly contributing to drug retention (142, 143). It is worth noting that some tumors do not have increased vascularity, and therefore the EPR effect is not observed in them. For example, metastatic prostate and liver cancers have low vascular densities (144, 145). This low vascular density potentially explains the lack of FLS-, ICG-, and 5-ALA-labeling of some brain metastases.

In order for the EPR effect to occur, the injected molecule should be biocompatible, have no clearance by the reticuloendothelial system, and be non-reactive to blood cells or the endothelium (142). The molecule should be large enough (>40 kDa) to avoid renal clearance through the pores in glomerular endothelium and have a weakly negative or neutral surface charge (142, 146). With respect to the upper limit of size of a molecule for EPR, researchers have demonstrated that 1-μm diameter *Lactobacilli* can be selectively delivered into the tumor with additional dilation of the tumor endothelial cell junctions by an angiotensin-converting enzyme inhibitor (147). Many drugs, including polymer conjugates, bind to albumin (60 kDa), increasing their molecular weight, thus satisfying the criteria for EPR delivery. For example, immunoglobulin G (160 kDa), polymer-drug conjugates, and liposome-encapsulated drugs fit the above-mentioned criteria.

Distinct accumulation of a high-molecular-weight drug via EPR can be seen within half an hour, with a maximum tumor-to-normal tissue ratio within hours to days after administration (142). The retention time usually ranges from hours to days. In comparison, low-molecular-weight contrast agents, including gadolinium-based contrast agents (500–1,000 Da in size) are capable of freely diffusing through the peripheral endothelium (148). These agents accumulate within the tumor because of a first-pass effect, but are not retained. Thus, compared to smaller molecules, the model EPR macromolecule is small enough to enter endothelial pores of the abnormal tumor vasculature, where it more preferentially accumulates, but large enough to avoid renal clearance, allowing prolonged circulation (142, 143).

Of note, all current clinically employed optical tracers for brain tumor imaging (FLS, ICG, and 5-ALA) rely to some degree on the EPR effect to exit the tumor microvasculature through a compromised BBTB.

### BBB Disruption

#### Hyperosmolar Opening

**Figure 4:**
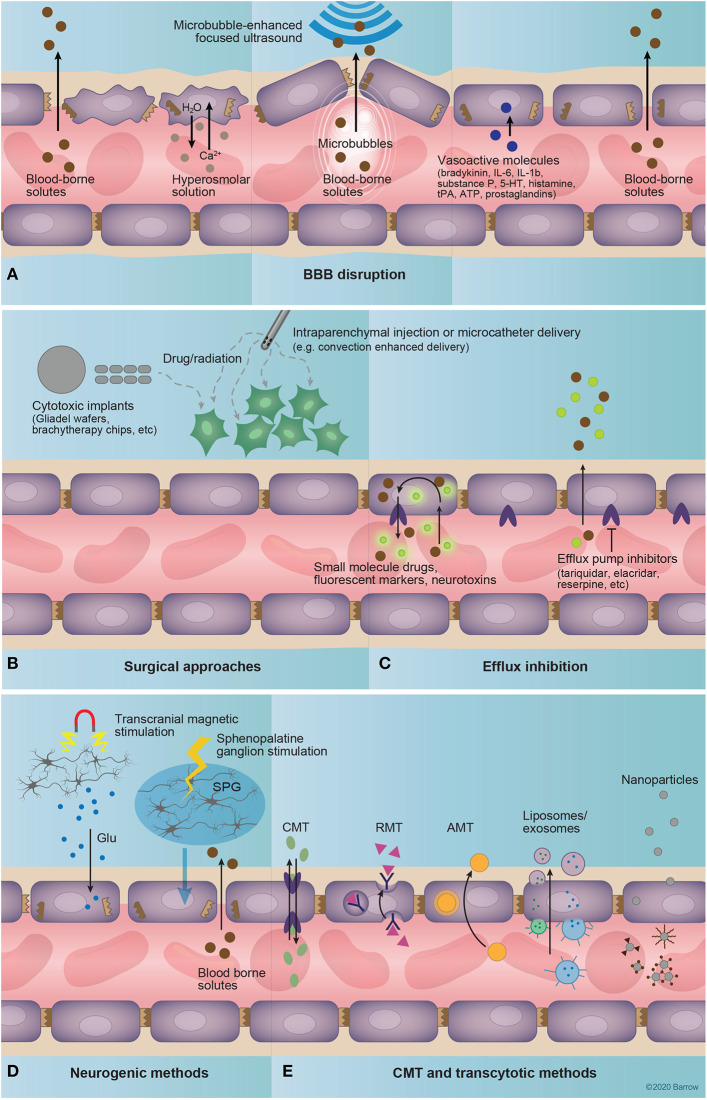
Drug delivery to the central nervous system. **(A)** Techniques that involve coadministration of a drug coupled to disruption of the blood-brain barrier (BBB) include hyperosmolar disruption, microbubble-enhanced focused ultrasound (MEUS), and administration of vasoactive substances. The intravascular injection of a hyperosmolar solution dehydrates endothelial cells, shrinking them and causing strain at tight junctions, and ultimately causing tight-junction protein displacement and BBB disruption. MEUS involves the intravascular administration of small (~1 μm) bubbles that are targeted by focused ultrasound to induce cavitation, creating tight-junction strain and resulting in BBB permeation. Finally, vasoactive molecules including bradykinin, interleukins 6 and 1β, substance P, serotonin, histamine, tissue plasminogen activator, adenosine triphosphate, and prostaglandins, alter the expression of tight-junction proteins, affecting BBB permeability. **(B)** Surgical approaches deliver drugs by circumventing the BBB entirely. These approaches include the implantation of a polymer with delayed drug release [for example, radioactive brachytherapy (157) or chemotherapeutic-loaded implants (157, 158)] into the brain or the injection of a drug into the brain via microcatheter. **(C)** Efflux inhibition aims to augment the diffusion of a drug across the BBB via the inhibition of efflux pumps that mediate its active removal from the brain. Examples of known efflux pump inhibitors include tariquidar, elacridar, and reserpine (159). **(D)** Neurogenic methods of increasing BBB permeability include transcranial magnetic stimulation and sphenopalatine ganglion stimulation. Transcranial magnetic stimulation is thought to increase BBB permeability through the endothelial binding of glutamate released from magnetically excited neurons. The exact mechanisms of sphenopalatine-mediated BBB permeability are currently unknown, but likely involve sympathetic innervation of intracranial vasculature provided through the sphenopalatine ganglion. **(E)** Carrier-mediated and transcytotic methods involve the targeted binding of drug-bearing molecules to structures expressed on the luminal surface of the endothelium. This delivery system includes freely soluble drug molecules that bind to expressed transporters or surface molecules, drug-delivery vehicles such as liposomes or exosomes, or nanoparticles. Importantly, these drug-delivery methods often incorporate transporter or receptor substrates or molecular probes for targeting to specific structures at the BBB/BBTB. 5-HT, serotonin; AMT, adsorption-mediated transport; ATP, adenosine triphosphate; BBB, blood-brain barrier; CMT, carrier-mediated transport; Glu, glutamate; IL, interleukin; RMT, receptor-mediated transport; SPG, sphenopalatine ganglion; tPA, tissue plasminogen activator. Used with permission from Barrow Neurological Institute, Phoenix, Arizona.

Focus on drug delivery via disruption of the BBB began in the 1970s by Rapoport (149) and Rapoport et al. (150), establishing the effectiveness of temporary TJ opening with intraarterial infusion of a bolus of hyperosmolar mannitol (Figure 4A) (151). This method was subsequently adapted to increase the delivery of chemotherapeutics to brain tumors (152, 153). Although there are reports on the safety and efficacy of the selective endovascular hyperosmolar opening of the BBB for chemotherapeutics in cases of CNS lymphoma, anaplastic oligodendroglioma, and other brain malignancies (154, 155), overall effectiveness of this method is still debatable (91). The non-selective opening of the BBB allows for indiscriminate influx of blood-borne molecules, causing neurotoxicity, vasculopathy, seizure, and chronic neurologic defects, which limit its widespread clinical use (20, 91, 156). However, since the inception of this idea, there has been considerable advancement in other techniques to disrupt the BBB for theranostic applications.

#### Microbubble-Enhanced Focused Ultrasound

Microbubble-enhanced focused ultrasound (MEUS) techniques rely on the application of low-power ultrasound to a specific brain region in combination with intravenous (IV) administration of preformed, lipid-coated, echogenic microbubbles (Figure 4A) (160). The focused ultrasound induces stable cavitation in the bubbles as they pass through the ultrasound field, mediating precise and transient disruption of the BBB via the downregulation of TJ proteins (82), suppression of P-glycoprotein expression (161), and facilitation of pinocytosis (162). MEUS has been used recently in animal models to enhance BBB permeability to an array of therapeutics (160). Furthermore, this technique can be used under magnetic resonance imaging (MRI) guidance to evaluate the effectiveness of BBB opening. Such a combination has been explored clinically for targeted neuromodulation (163) and drug delivery in Alzheimer's disease (164). MEUS is designed for a non-invasive and highly targeted approach, mitigating the risks of non-specific hyperosmolar BBB disruption and surgical methods of drug delivery (165, 166).

#### Neurogenic Methods

Transcranial magnetic stimulation and sphenopalatine ganglion stimulation (Figure 4D) are novel methods that can affect neurovascular unit function and BBB permeability by acting on its neuronal component. Transcranial magnetic stimulation effects are based on neuronal activity and are mediated by an N-methyl-D-aspartate-receptor-dependent mechanism (167, 168). Transcranial magnetic stimulation has been used for the treatment of neurological disorders, including depression, but its potential for improving drug delivery to the brain has become a subject of recent studies (167). Sphenopalatine ganglion stimulation is a novel technique for transient (4 h) BBB disruption via neural stimulation (57). Sphenopalatine ganglion stimulation caused downregulation of TJ proteins, similar to that seen in hyperosmolar disruption, and resulted in ipsilateral, hemispheric extravasation of a 70-kDa FLS-labeled dextran in a rat model (57). The safety and clinical efficacy of this method, especially compared to the more focused MEUS, is yet to be established.

#### Photodynamic Method

Photodynamic opening of the BBB using 5-ALA has been described in a mouse model (169). Irradiation with a 635-nm laser, 30 min after IV administration of 5-ALA resulted in a transient (4 h) increased BBB permeability to Evans blue dye, 70-kDa FITC-dextrans, and intravascular solutes. Histological analysis demonstrated complete recovery from the induced perivascular edema within 3 days. Implications of these findings for the current 5-ALA-based fluorescence-guided surgical techniques or photodynamic therapy should be investigated further. However, 5-ALA is mostly cleared from the blood by the time of glioma resection (~6 h after oral administration).

#### Clinical Applicability of BBB Disruption for Fluorescence-Guided Surgery

With regard to optically guided brain tumor resection, techniques for BBB disruption may be used to enhance the delivery of targeted optical markers, improving the sensitivity of tumor visualization, especially in low-grade gliomas and at the border of high-grade gliomas, where the BBTB remains largely intact. However, clinical procedures for BBB disruption may be too invasive and time consuming to warrant their application solely to improve fluorescence-guided surgery. Preoperative BBB disruption for delivery of targeted optical drugs should ideally be combined with therapeutic agents in order to take full advantage of transient BBB opening. Further, wider adoption of MEUS and transcranial magnetic stimulation should stimulate the clinical translation of novel combinatory drugs that include not only therapeutic agents, but also optical fluorescent tracers for better intraoperative visualization of brain tumor margins. However, major efforts are directed toward development of molecular techniques for bypassing and targeting the BBB/BBTB without destroying its integrity (170).

### Bypassing the BBB

Techniques to bypass the BBB entirely are primarily surgical in nature (Figure 4B). These include methods such as intrathecal/intraventricular injection, transnasal administration, convection-enhanced delivery (CED), and intracerebral or topical implantation.

#### Intrathecal or Intraventricular Administration

Intrathecal or intraventricular administration theoretically bypasses the BBB, but diffusion from the cerebrospinal fluid (CSF) to the brain parenchyma is limited by a relatively rapid bulk flow of CSF and a slow rate of diffusion into brain tissue (87). Thus, drugs administered to the CSF are ultimately redistributed to the blood where they must cross the BBB to be effective. While intrathecal or intraventricular drug delivery is effective for leptomeningeal diseases, it is not suitable for parenchymal brain tumors (64, 171).

#### Transnasal Drug Administration

Transnasal drug administration and mucosal engrafting (172, 173) techniques initially avoid the BBB, but the drug must still cross into the subarachnoid space, where it will face the same obstacles as intrathecally administered drugs while providing only limited focal drug distribution.

#### Convection-Enhanced Delivery

CED involves surgical insertion of a semipermeable catheter into the area of drug administration in the brain or tumor with a constant administration of drug solution under a positive-pressure gradient (174, 175). Over 20 clinical studies used CED for delivery of therapeutic agents in high-grade gliomas and showed moderate clinical efficiency (158). A few studies have used coinfusion with gadolinium to monitor infusate distribution (176, 177). Wang et al. (93) investigated CED of an ^18^[F]-positron-emitting, fluorescent derivative of the Abl-kinase inhibitor dasatinib in a mouse glioma model. Similar to other studies (178, 179), the main purpose of coadministration of the fluorescent agent is to monitor drug distribution in experimental settings. As trials for the delivery of novel therapeutics using CED are still being conducted (180, 181), the utilization of CED with fluorescent markers for optical image guidance, for example in recurrent gliomas, is yet to be explored.

Another method for circumvention of the BBB is to deliver drug directly into the resection cavity intraoperatively. This technique has seen relative success with current therapeutic agents, such as an implantable, biodegradable polyanhydride polymer infused with the alkylating agent carmustine (Gliadel) (182–184) or a collagen wafer embedded with x-ray-emitting cesium-131 (94). Such administration promotes delayed diffusion of the drug that creates areas of increased drug concentration and CED in the peritumoral bed over time. Direct intracavitary delivery of optical imaging agents, especially “activatable” fluorescence probes that turn on upon specific binding to a targeted molecular motif, is also being explored (185, 186). Although promising, local application of fluorescent markers to the surface of a resection cavity is inherently limited in that it requires some incubation time and would leave any subsurface tumor unlabeled. On the other hand, topical staining could still be useful to identify any apparent residual tumor. Furthermore, such staining could be used for a small-field, intraoperative, digital biopsy assessment of selected regions of interest (187, 188).

### Targeting the BBB

Recent technologies have adopted a strategy of targeting existing transporters, receptors or other molecules expressed on the luminal surface of the CNS endothelium to facilitate drug delivery (Figures 4C,E). Importantly, these techniques allow for crossing of the BBB without disrupting interendothelial TJs, thereby avoiding the potential efflux of neurotoxic substances from the blood into the brain.

#### Carrier-Mediated Transport

Carrier-mediated transport (CMT) takes advantage of an array of small-molecule transporters expressed at the BBB. Through the conjugation of small-molecule drugs to or the mimicking of the ligands of these transporters, selective movement of drugs across the BBB may be achieved. Classically, drugs such as L-3,4-dihydroxyphenylalanine, melphalan, and gabapentin take advantage of CMT for CNS activity. Singh and Subudhi (116) have demonstrated this by delivering a methotrexate-lysine conjugate prodrug across the BBB via L-type amino acid transporter 1. In a similar approach, Peura et al. (117) synthesized amino acid prodrugs of dopamine to increase uptake across the BBB via the large amino acid transporter 1. While these early results have been successful in animal models, clinical application of CMT-based delivery may be difficult. Examination of the expression profiles of these receptors in a given tumor or BBTB might be needed prior to determination of a therapeutic target. Further, CMT-based strategies are limited to small-molecule drugs or prodrugs, as transporters will not support the passage of larger molecules.

#### Receptor-Mediated Transport

Receptor-mediated transport (RMT) strategies target receptors expressed on the luminal surface of CNS endothelium in order to initiate endocytosis or transcytosis for transport of the molecule to the abluminal surface. Often, these strategies involve the linking of an effector molecule to a receptor ligand or antibody that can bind and initiate endocytosis.

An example of a molecule used successfully for RMT is angiopep-2 (ANG2), a synthetic peptide that targets LRP1 at the BBB. Importantly, LRP1 is a shuttling receptor, able to mediate transcytosis while avoiding the destructive lysosomal compartment (111, 189). LRP1 has also been shown to be overexpressed on glioblastoma cells (190), giving ANG2 tumor-specific targeting capabilities. So far, ANG2 has proven capable of delivering paclitaxel to glioma and brain metastases, avoiding P-glycoprotein-mediated paclitaxel efflux (90), and is currently in Phase II clinical trials for recurrent high-grade glioma (NCT01967810) and breast cancer with recurrent brain metastases (NCT02048059).

Rajora et al. (118) reported the successful targeting of LRP-1 expressed on orthotopic U87 glioblastoma cells in mice using apolipoprotein E3 porphyrin-lipid nanoparticles. Glioblastoma cells readily took up the nanoparticles, exhibiting near-infrared fluorescence and sensitization to photodynamic therapy. In another study, Lam et al. (119) reported the success of transferrin-functionalized nanoparticles bearing temozolomide and bromodomain inhibitor JQ1 to selectively target and kill glioblastoma cells in a mouse model. These studies both demonstrate the efficacy of RMT-mediated targeting of tumors *in vivo*. Importantly, these therapies have a potential to traverse the intact portion of the BBTB for treatment of otherwise shielded tumor cells.

#### The Future and Caveats of Techniques for Transcellular Transport

Increasingly, CMT- and RMT-based therapeutic strategies are incorporating delivery vehicles such as nanoparticles, liposomes, or exosomes to target brain tumors. These strategies involve the assembly of a custom-built molecular vehicle, often housing an effector molecule, that is coated with receptor ligands or other targeting molecules to facilitate the precise targeting of the vehicles to the endothelium of the BBB or tumor cells. A detailed review of liposome-based drug vehicles was recently published (141), and several have been published regarding nanoparticle-based drug delivery to the brain (85, 89, 170, 191–196). The main advantage of nanoparticle technology is its customizability, as the size, composition, cargo, and target are tunable, affecting changes in BBB permeability, therapeutic effect, or drug pharmacokinetics. However, several caveats to these strategies need to be carefully considered.

Bypassing the endothelial barrier with an optical or other diagnostic agent only informs about the location and degree of BBB disruption and not necessarily about the tumor cells themselves. In order to achieve better selectivity and ability to target tumor cells beyond the competent BBTB, the agent should dissociate from the encapsulating vehicle, travel through the extracellular space and, ideally, label the tumor cells. Given the complexity of the underlying mechanisms of drug delivery, many obstacles must be overcome. If the vehicle is too stable, the drug is not released and does not react with the target (197, 198). However, if the drug has low molecular weight, it risks diffusion back to the circulation upon dissociation from the large carrier (197, 199, 200). Large molecular constructs, and especially micelles, should additionally withstand shear stress of the microvascular circulation (201). For example, it has been demonstrated that a significant amount of doxorubicin has leaked out of encapsulating micelles within a few hours after administration because of shear stress (200). These problems are only a few considerations that hinder the safety and efficacy of these therapies, but nonetheless, they must be reconciled before clinical application.

## Mechanisms of Fluorescent Labeling of Brain Tumors

### 5-Aminolevulinic Acid

#### General Characteristics

**Figure 5:**
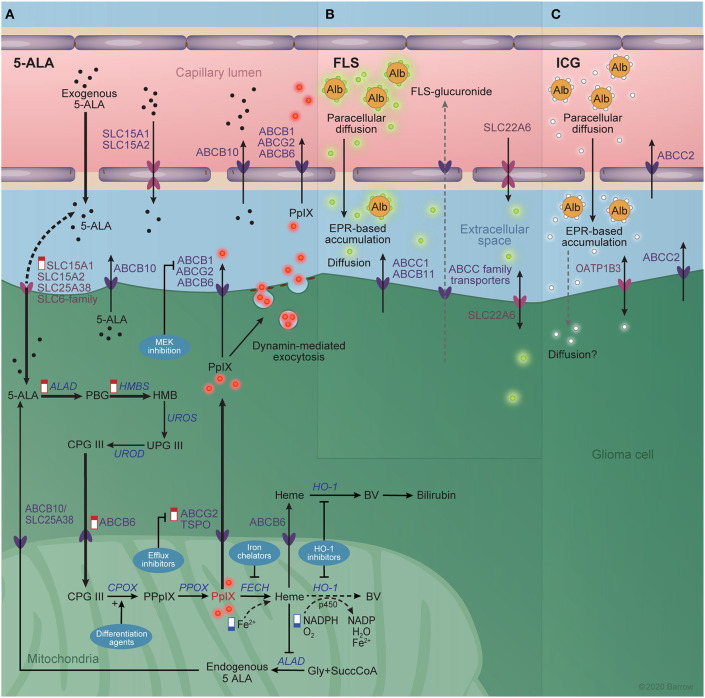
Interaction between imaging agents and the blood-brain barrier. **(A)** The known transporters of 5-aminolevulinic acid (5-ALA) and protoporphyrin IX (PpIX) at the blood brain barrier and glioma cell membrane are shown schematically. The heme biosynthetic pathway is shown in the glioma cell cytoplasm and mitochondrial matrix. Alterations in the heme biosynthesis pathway responsible for increased PpIX accumulation in the tumor are indicated by “low” (blue) and “high” (red) signs. Thick arrows represent increased rate reactions after exogenous 5-ALA administration that contribute to the excess PpIX accumulation. Dotted arrows represent reduced rate reactions contributing to PpIX accumulation. Techniques for augmenting 5-ALA-mediated PpIX accumulation are shown in blue bubbles and include MEK inhibition, efflux inhibitors, differentiation agents, iron chelators, and heme oxygenase 1 inhibitors. **(B)** The known transporters of fluorescein and **(C)** indocyanine green are shown similarly. Active efflux pumps of the ATP-binding cassette family are depicted in dark violet and solute carrier family transporters in pink. Alb, albumin; ABC, ATP-binding cassette; ALAD, aminolevulinic acid dehydrogenase; BV, biliverdin; CPG III, coproporphyrinogen III; CPOX, CPG III oxidase; FECH, ferrochelatase; Gly, glycine; HO-1, Heme Oxygenase 1; HMB, hydroxymethylbilane; HMBS, HMB synthase; PBG, porphobilinogen; PpIX, protoporphyrinogen IX; PPOX, PpIX oxidase; SLC, solute carrier; SuccCoA, Succinyl Coenzyme A; UPG III, uroporphyrinogen III; UROD, UPG III decarboxylase; UROS, UPG III synthase. *Used with permission from Barrow Neurological Institute, Phoenix, Arizona*.

5-ALA (C_5_H_9_NO_3_, 131.13 Da), is an endogenous metabolite and a precursor for the biosynthesis of heme *in vivo* (Figure 5A). Under the action of intracellular enzymes in the heme biosynthetic pathway, 5-ALA is converted to an endogenous fluorophore, protoporphyrin IX (PpIX) (excitation: 405 nm; emission: 635 nm, with a minor emission peak at 710 nm). Intraoperatively, the blue excitation light contrasts well with the red fluorescence of PpIX, facilitating tumor margin delineation. 5-ALA was initially investigated for use in photodynamic therapy (202–205), whereby absorption of light by PpIX mediates the production of tumor-killing reactive oxygen species, but became a drug for improved visualization of high-grade gliomas during resection (206, 207) approved by the US Food and Drug Administration (FDA) (208). The mechanisms of 5-ALA passage through the BBTB (209) and intracellular metabolism (127, 210) have been previously reviewed in detail, so here we provide a brief summary of the key steps.

#### 5-ALA Delivery Across the BBTB

The oral bioavailability of 5-ALA is around 60% in healthy subjects (127). Plasma half-life of 5-ALA after oral administration is about 45 min and plasma concentration approaches zero after about 6.5 h (211, 212). Experiments with radioactive 5-ALA in mice showed that 5-ALA does not penetrate an intact BBB but accumulates and undergoes conversion to PpIX in brain regions with a diminished BBB, such as the ependyma, choroid plexus, arcuate nucleus, and median eminence. (213, 214) PpIX visualization in high-grade gliomas correlates well with gadolinium-enhanced MRI (215), suggesting that the main mechanism of accumulation of PpIX in glioma is leakage of free 5-ALA through the altered BBTB. The lack of PpIX accumulation in low-grade gliomas is likely due to the presence of a more competent BBTB that prevents 5-ALA delivery and a lack of severe alterations in heme metabolism that would result in PpIX accumulation. So far, it is hard to elucidate which mechanism is most determinate of 5-ALA accumulation. Observed foci of fluorescence in low-grade gliomas were correlated with anaplastic transformation and increased cellularity (216, 217), which in turn may result in a less competent BBTB that is permeable to 5-ALA but not to gadolinium-based contrast.

#### 5-ALA Metabolism

Once inside the cell, 5-ALA enters the heme biosynthetic pathway, where it is converted sequentially to porphobilinogen, hydroxymethylbilane, uroporphyrinogen III, coproporphyrinogen III, protoporphyrinogen IX, PpIX, and heme (Figure 5A). Peak fluorescence intensity of PpIX in the tumor core is observed about 7 to 8 h after oral 5-ALA administration, while in the marginal tumor area the peak of weaker fluorescence is observed after about 8 to 9 h (218, 219).

In normal non-erythroid cells, heme biosynthesis is regulated by negative feedback mechanisms, mainly negative feedback of heme on the enzyme ALA synthase, controlling the intracellular levels of PpIX (220). In cancer cells, however, these systems are dysregulated, leading to PpIX accumulation and fluorescence. A recent examination of various human tumor samples suggests that the kinetics of PpIX accumulation are determined primarily by alterations in PpIX efflux, conversion of PpIX to heme, and PpIX biosynthesis (221). Interestingly, fluorescence was not shown to be dependent on the rate of 5-ALA uptake (221).

Heterogeneity in tumor cell signaling, protein expression, and metabolism are responsible for variations in PpIX accumulation. In this way, differential expression of transporters for elements of the heme biosynthetic pathway at the BBTB or tumor cell membrane is a source of modulation of the accumulation of PpIX. Notably, 5-ALA is a substrate of SLC15A1 and SLC15A2 (222), SLC36A1 (223), SLC6A6 and SLC6A13 (224), and ABCB10 (210), while PpIX is a substrate of ABCB6, ABCG2 (225) and ABCB1 (126). Of these, SLC15A1, SLC15A2, ABCB10, ABCB1, ABCG2, and ABCB6 are known to be expressed at the BBB (226), and therefore likely influence levels of PpIX accumulation. Kitajima et al. (227) showed that dynamin 2-mediated exocytosis plays a role in PpIX efflux from cancer cell lines in the JFCR39 panel, which includes six brain tumor lines. Interestingly, they failed to show a correlation between ABCG2 expression levels and PpIX efflux, although ABCG2 inhibition led to enhanced PpIX accumulation. This is in contrast to previous results by Hagiya et al. (228) that showed ABCG2 expression to be a major determinant of PpIX fluorescence in gastric cancer cell lines *in vitro*.

#### PpIX Excretion

PpIX may be converted to heme in mitochondria via the action of ferrochelatase or may be subject to active efflux from the cell by the ABCG2 efflux pump. Once in the blood, the plasma half-life of PpIX is around 8 h (229). If not further metabolized to heme, PpIX is taken up and excreted into bile by the liver (210).

#### Approaches to Augment PpIX Accumulation

The complex metabolism and transport of PpIX precursors create opportunities for the pharmacologic augmentation of PpIX accumulation in tumors that bear relevance for photodiagnostic and photodynamic applications of PpIX. At least five mechanisms have been identified (Figure 5A).

##### Mitogen-Activated Protein Kinase Kinase (MEK) inhibition

Yoshioka et al. (126) demonstrated enhancement of PpIX accumulation in a murine mammary tumor model via the inhibition of oncogenic Ras/MEK. MEK inhibition decreased expression of the ABCB1 efflux pump and reduced activity of ferrochelatase, the enzyme responsible for converting PpIX to non-fluorescent heme. Importantly, these changes were not observed in normal tissues, highlighting a targetable difference in regulatory mechanisms for heme biosynthesis between tumor and normal cells.

##### Efflux inhibition

Efflux pump inhibition relies on the principle that a known set of targetable transporters are responsible for the efflux of PpIX or its precursors from the brain or tumor tissues (Figure 4C). Kawai et al. (230) demonstrated that expression of the efflux pump ABCG2 was associated with a cancer stem cell phenotype and decreased 5-ALA fluorescence in a human pancreatic cancer cell line. Studies in peripheral tumors (127, 128) and with tumor cells *in vitro* (129, 130) share these results, showing an increase in 5-ALA-mediated PpIX accumulation in tumor tissue when combined with ABCG2 inhibitors. However, these studies do not necessarily account for the added heterogeneity of transporter expression at the BBB or within tumors *in vivo*.

##### Iron chelators

Augmentation of PpIX fluorescence by ferrochelatase inhibition using iron chelators has been demonstrated *in vitro* in glioma cell cultures (131–133). Wang et al. (134) showed that glioma stem cell escape from PpIX fluorescence could be overcome with the use of iron chelation to inhibit ferrochelatase, but not with administration of an ABCG2 inhibitor. In fact, contrary to the abovementioned reports, ABCG2 inhibition was shown to slightly decrease PpIX accumulation in these cells. The authors suggest that these results are likely due to the off-target inhibition of ABCB6, required for trafficking of coproporphyrinogen III across the mitochondrial membrane, leading to decreased PpIX synthesis overall. These results emphasize the importance of targeting specific transporters if efflux inhibition is to be employed and also raise questions about the viability of this approach in all tumors.

##### Differentiation agents

Differentiation therapy (calcitriol, vitamin E) has been shown to increase the cytotoxic effects of 5-ALA-mediated photodynamic therapy via upregulation of coproporphyrinogen oxidase in epithelial cancers (135, 137). Calcitriol was also shown to enhance 5-ALA-induced fluorescence and photodynamic therapy in human glioma cells *in vitro* (136).

##### Heme oxygenase 1 (HO-1) inhibition

Inhibition of HO-1 has also been shown to increase PpIX accumulation in melanoma (138). This strategy has the added benefit of disabling the cytoprotective and anti-inflammatory properties of HO-1, making tumor cells more prone to lysis by oxidative stress (139).

#### Opportunities and Limitations of 5-ALA Augmentation

Overall, the intricacy of cell-signaling pathways and transporter expression that governs heme biosynthesis complicates PpIX-based fluorescence visualization. Notably, elements of the heme biosynthetic pathway are substrates of a diverse set of transporters that modulate their intracellular accumulation. Furthermore, heme biosynthetic enzymes are often aberrantly expressed or active in tumor cells, hampering the reliable prediction of PpIX fluorescence status. For example, Lai et al. (231) demonstrated that selective inhibition of SLC15A1 and SLC36A1 expressed in normal but not cancerous lung and prostate cell lines allowed for augmentation of PpIX accumulation in tumor cells. This approach may actually hinder PpIX accumulation in glioma, though, as SLC15A1 is expressed at the BBB and may mediate 5-ALA accumulation in the brain (231). Nevertheless, the general approach of inhibiting transporters selectively expressed in normal cells versus tumor cells may still prove useful to augment tumor-selective accumulation of PpIX.

The described approaches provide tools for control of the selective augmentation of PpIX fluorescence in tumor cells. While these results remain to be evaluated amidst the added complexity of the BBB and BBTB in humans, they support the viability of pharmacological strategies to enhance PpIX fluorescence. Further research may one day see the ultimate translation of these approaches to the operating room, where they can help to augment the sensitivity and utility of 5-ALA-guided brain tumor resection.

### Fluorescein Sodium

#### General Characteristics

FLS (C_20_H_10_Na_2_O_5_, 376.5 Da) is a yellow-green fluorescent dye (excitation 460–490 nm, emission 510–530 nm) (232) whose derivatives, such as FITC and Alexa 488, are used widely in research. The LD_50_ of IV FLS is 300 mg/kg in rabbits (233). FLS-guided resection of high-grade glioma was successful in a recent Phase II clinical trial (*n* = 46 patients) (234) and in a prospective observational study (*n* = 279 patients) (78), indicating its clinical applicability. FLS has also been used as a contrast for intraoperative assessment of tissue microstructure using confocal laser endomicroscopy (235, 236).

#### Pharmacokinetics

After IV administration, FLS weakly binds to serum albumin and rapidly distributes throughout the central circulation. FLS has a volume of distribution of 0.5 L/kg (237), indicating an even distribution between blood and interstitial spaces. FLS exists in the circulation in both a free unbound fraction and a serum-protein bound fraction. The small size of unbound FLS allows for rapid diffusion into the tumor due to first-pass extravasation, creating a large early peak in tumor fluorescence (Figure 5B). In contrast, the larger effective diameter of protein-bound FLS leads to a later smaller peak that is facilitated by the EPR effect. The unbound fraction of FLS only minimally crosses the intact BBB when administered in high doses (95) but is not readily detected within the normal brain using the operative microscope at the time of surgery. Unspecific leakage of unbound FLS through a heterogeneous and dynamic BBB/BBTB explains reports of unspecific FLS staining in peritumoral areas (238). Compared to unbound FLS, a PEGylated form of FLS with a molecular weight similar to that of gadolinium-based contrast agents (939 Da) showed improved specificity of accumulation in U251 orthotopic mouse gliomas 1 h after administration (95). An albumin-bound formulation of 5-aminofluorescein (66,950 Da) was investigated in a Phase I/II study in Germany (96, 97), where bright fluorescence was observed in 10 of 10 patients with high-grade gliomas 0.5 to 4 days after IV administration. These results highlight the utility of the EPR effect for prolonging the fluorescent staining window for small non-targeted molecules.

#### Optimal FLS Timing and Dosage

Increasing the dose of FLS (5 vs. 20 mg/kg human equivalent) results in an increase in unbound FLS in the blood and normal brain tissue (95). Therefore, the dose and timing of FLS administration should be considered to optimize fluorescent contrast during surgery. Thus far, the optimal strategy is to use lower doses of FLS [1–2 mg/kg (239), 3 mg/kg (240), and 5 mg/kg (234)] to minimize unspecific extravasation and to administer FLS 2 to 4 h before visualization, which corresponds to the wash-out period of the FLS from the normal brain (78).

#### FLS and BBTB Heterogeneity

FLS tends to accumulate in extravascular spaces and not within tumor cells. Accordingly, FLS-defined high-grade glioma margins correlate well with gadolinium-based MRI results (240). Reports on the use of FLS for the visualization of brain metastases demonstrated that the majority of them were fluorescent [90/95 (95%) (241) and 16/17 (94%) (242)]. However, surrounding normal brain also showed a low degree of fluorescence (241). Data on FLS efficacy in low- and intermediate-grade gliomas show that about half of tumors are not labeled with FLS, likely because the BBTB is not sufficiently disrupted (243). However, some non-gadolinium-enhancing gliomas can still be labeled with FLS (244, 245). These findings support the hypothesis that the unbound, low-molecular-weight fraction of FLS plays an important role in labeling tumors with minimally disrupted BBTB. Such tumors and peritumoral brain regions may allow extravasation or convection-based transport of the smaller unbound FLS molecules but not gadolinium-based contrast. Thus, a balance must be struck between dosing that creates an increased proportion of unbound FLS that is more likely to unspecifically stain normal brain but more readily stain marginal tumor and dosing in which a smaller dose of free and protein-bound FLS promotes EPR-based labeling of the tumor core but less labeling of the surrounding normal brain.

#### Potential Molecular Transporters and Clearance of FLS

Molecular transporters may also play a role in FLS pharmacokinetics. In the liver, FLS is conjugated to glucuronide before being excreted in the urine. FLS has a plasma half-life of 23.5 min, while minimally fluorescent FLS-glucuronide has a plasma half-life of 264 min (237). The small size and relative lipophilicity of FLS may allow for greater BBB permeability compared to ICG or gadolinium-based agents, but the activity of certain transporters at the BBB or BBTB may limit unaided extravasation. FLS is a substrate for the SLC22A6 transporter (237), the bile salt export pump, ABCB11 (246), and multidrug resistance-associated protein 1 (ABCC1) (247). Notably, SLC22A6 is involved in drug clearance from the CSF (248) and is expressed at low levels in CNS endothelium (226), and ABCC1 is expressed at the BBB (25). FLS-glucuronide is also likely a substrate of other members of the ABCC-subfamily of multidrug resistance proteins that are responsible for transporting conjugated metabolites (249), although, to our knowledge, this has not been shown. As such, these transporters likely alter the BBB permeability of unbound FLS.

### Indocyanine Green

#### General Characteristics

ICG (C_43_H_47_N_2_NaO_6_S_2_, 774.96 Da) is a water soluble, unstable [half-life in aqueous solution is 20 h (250)], amphiphilic fluorescent dye. The LD_50_ of IV ICG is 50 to 75 mg/kg (251). After IV injection, ICG rapidly binds to plasma proteins and is distributed throughout the circulation with a volume of distribution of 0.035 L/kg (252), reflecting a high degree of plasma protein binding (140). The excitation and emission maximums of bound ICG exist in the near-infrared range (805 and 830 nm, respectively) thus enabling greater tissue penetration than markers that fluoresce in the visible range (253, 254).

#### Pharmacokinetics

ICG binding with plasma proteins increases the brightness of ICG fluorescence nearly 3-fold. Although it was initially thought that IV-injected ICG binds to albumin (255), later studies of blood samples following IV ICG injection suggested that ICG binds more intensely to high density lipoproteins (175–360 kDa, 7–14 nm) and moderately to low-density lipoproteins (256, 257). The larger size of bound ICG and the fact that, unlike FLS, ICG is almost completely bound to plasma proteins are important considerations for understanding the transport of ICG across the BBTB (Figure 5C).

Bound ICG gradually traverses the incompetent BBTB and accumulates in brain tumors such as high-grade gliomas and meningiomas, primarily due to the EPR effect (258). The timing of ICG accumulation in the tumor and optimal dosing of ICG was the subject of animal and human studies that could be grouped into three categories based on the imaging time: imaging immediately after ICG injection, delayed imaging within a few hours after injection, and imaging within a day after injection, termed the second-window ICG (251, 259, 260).

A study by Haglund et al. (251) investigated ICG in low- and high-grade gliomas using a charge-coupled device camera attached to a standard operating microscope within the first 10 min after IV ICG administration at 1 mg/kg. They observed progressively increased ICG signal within 10 min of recording in a high-grade glioma.

A study by Charalampaki et al. (261) demonstrated ICG accumulation in high-grade gliomas and meningiomas 1 h after IV injection of 50 mg ICG (<1 mg/kg). They used a commercially available operating microscope with a near-infrared imaging mode (ARveo Glow800, Leica microsystems, Wetzlar, Germany). In a study by Eyüpoglu et al. (262), 5 mg/kg ICG was administered intravenously at the end of high-grade glioma resection. Using this technique and a standard operating microscope (OPMI Pentero, Carl Zeiss AG, Oberkochen, Germany) the authors were able to highlight PpIX-negative, hypervascularized transitional tumor zone (262).

Several studies (98–102) assessed high-dose ICG (5 mg/kg administered IV over 1 h, 24 h prior to surgery) with a dedicated commercial near-infrared exoscope/endoscope imaging system (Iridium, VisionSense, Medtronic, Dublin, Republic of Ireland) in various brain tumors to take advantage of EPR-based drug accumulation.

#### ICG and BBTB Heterogeneity

It is worth noting that in all three ICG-based tumor-imaging methods described, high-grade gliomas were efficiently labeled with ICG. In low-grade gliomas, fluorescence intensity returned to the baseline within 5 min after a slight delay in clearance compared to the adjacent normal brain (251). Extravascular ICG in the tumor region does not specifically bind to brain tumor cells; however, it does penetrate certain cells, creating cytoplasmic contrast for confocal imaging and histopathologic tissue analysis (261, 263).

#### Potential Molecular Transporters and Clearance of ICG

ICG is rapidly and exclusively cleared by the liver. There is evidence in animal models that ICG clearance proceeds in a biphasic method, with an initial phase of rapid clearance resulting in a half-life of 2 to 4 min and a secondary phase with a half-life of more than an hour at low concentrations (264–266). Uptake into hepatocytes and excretion into bile is mediated by two members of the ABC transporter family: the canalicular multispecific organic anion transporter 1 (ABCC2) and the bile salt export pump (ABCB11) (267, 268).

There is also evidence that ICG-uptake is enhanced in peripheral hepatocellular carcinoma cells expressing organic anion transporting polypeptide 1B3 (SLCO1B3), a member of the SLC family of membrane transporters (269). Importantly, ABCC2 is expressed at the BBB (27), and so may mediate the active efflux of ICG. While SLCO1B3 and ABCB11 are not significantly expressed at the BBB, their aberrant expression in tumor cells would likely modulate the dynamics of ICG-staining, warranting investigation of their expression if ICG visualization is to be used.

## Optimizing Delivery of Optical Agents to Brain Tumors Across the BBB and BBTB

Various fluorophores, including currently approved ICG and FLS, could be conjugated to other molecules or packed in molecular vehicles with two main goals: first, to improve tumor-to-normal brain contrast by increasing delivery efficiency compared to a fluorophore alone and, second, to improve the specificity of delivery by including targeting mechanisms. Here, we discuss the vehicles irrespective of the optical agent, assuming that criteria and considerations for optical agent selection are a completely separate issue mostly related to the detection methodology and tools. These optical considerations were reviewed previously (270–273). It is worth noting that any study of novel molecular vehicles, even for non-imaging purposes of brain tumor treatment, is relevant to the development of fluorescence-guided surgical technology and strategy because localization experiments are necessarily involved as part of the study. Among the available localization methods that include electron microscopy, radioactive tracers, and MRI, optical imaging methods and fluorescence microscopy are arguably among the most widely used and convenient approaches.

### Creating Small-Size Molecules

Efforts in nanotechnology research have been directed toward creating molecules small enough to pass the BBB and BBTB. Even if a molecule is designed that may pass the ~6-nm pore restriction in healthy BBB endothelium, it is unlikely that such a nanoparticle could achieve targeted delivery to tumor cells, as the size restriction greatly limits targeting options. However, several nanoparticles that are <100 nm in size have shown good tumor penetration in solid orthotopic animal gliomas.

One potential solution to this problem is quantum dots (QDs). QDs are nanoscale semiconductor crystals that can be excited to emit fluorescence. A unique property of QDs is that their absorption and emission wavelengths are functions of both the size and shape of the QD, allowing for the tailoring of emission and absorption spectra for desired applications (274). Tang et al. (103) report the use of aptamer-conjugated PEGylated QDs (~20-nm size) for the fluorescent visualization of epidermal growth factor receptor (EGFR)-expressing glioma. In this study, QDs were conjugated to a deoxyribonucleic acid oligonucleotide aptamer designed to bind to EGFR variant III (EGFRvIII), which is known to be expressed specifically on glioma cells. The authors showed that the QD-aptamer probe was able to cross the BBTB to highlight tumor cells in an orthotopic mouse model *in vivo*. Whether a nanoparticle of this size could pass the competent portions of a BBTB to label invading tumor cells *in vivo* still needs to be investigated.

Although QDs represent a novel class of nanoscale, highly stable, fluorescent molecules that have been stably conjugated to antibodies, peptides, and small molecule drugs (275, 276), several issues related to QDs specifically have to be addressed for clinical translation. Namely, stability within the pH-range in normal and tumor tissues, biosafety, stability within the circulation and clearance by the reticuloendothelial system are all characteristics that must be evaluated before clinical application. Furthermore, some QDs are composed of heavy metals including cadmium, lead, or mercury, which raises biosafety questions. While such QDs have yet to be tested clinically, successful preclinical studies warrant their further investigation.

### Enhancing EPR

Malignant tumors are notorious for disorganized and functionally abnormal vasculature that has sluggish blood flow, but which is a target for EPR optimization methods (142). The main strategies to improve permeability via EPR are directed toward dilating tumor vasculature, such as with nitric oxide-mediated vasodilation (angiotensin-converting-enzyme inhibitors, nitroglycerin, etc.), prostaglandin I2 agonists (105), tumor necrosis factor-a (106), or increasing systemic blood pressure by intraarterial administration of angiotensin II (107). The latter method improves tumor perfusion through unreactive tumor vessels that remain dilated while systemic vessels constrict and develop even tighter interendothelial connections. Such methods have commonalities with BBB disruption techniques and might be useful for enabling EPR through a relatively intact BBTB.

Increasing the molecular weight and size of a drug above 40 kDa by conjugating optical labels to targeting molecules or combining optical labels with large molecular vehicles would prolong drug circulation, increase the specificity of extravasation, and promote retention within high-grade gliomas and other tumors with severely disrupted BBTB for improved EPR-based drug delivery (277).

Xu et al. (104) developed silk fibroin nanoparticles loaded with ICG for imaging-guided photothermal therapy of orthotopic C6 glioma cells. Silk fibroin (200–350 kDa) is a biocompatible natural protein originating from silkworm cocoons. Conjugation with ICG created nanoparticles of about 200 nm in diameter, with improved EPR delivery in flank C6 gliomas, resulting in eight times higher intensity compared with free ICG injection 8 h after IV injection.

Nanoparticle delivery can enhance the circulation time and photostability of ICG (278) and offers a method for the specific targeting of ICG to tumor tissues. A drawback to this approach is limitation of drug delivery to the larger pores of the BBTB. Therefore, large molecules that are not targeted for transendothelial BBB or BBTB transport are unlikely to have better sensitivity in labeling glial tumors than already existing non-specific FLS, ICG, and metabolic 5-ALA.

### Tumor Targeting Coupled With Passive BBTB Transport

Molecular probes are molecules that bind specifically to a second target molecule, allowing for the interrogation of the properties of that molecule. Recent experimental studies have developed fluorescent molecular probes for labeling glioma. In general, fluorescent molecular probes are made up of two components: a signaling component (label) and an affinity component. The signaling component is a contrast agent or marker, such as a radionuclide, luciferin, or a paramagnetic molecule, that can generate a detectable signal, while the affinity component is a targeting molecule, such as an oligonucleotide or antibody, that specifically binds to and labels a molecule of interest. Importantly, the affinity component of these molecules is customizable, allowing for the precise targeting of minute differences in protein or cell-surface marker expression in tumor cells, thus providing contrast between tumor and normal tissue. These probes can be additionally classified based on structure, binding affinity, or type of signaling component (279, 280). Fortunately, advances in genome sequencing have allowed for better characterization of the aberrant genetic environment of gliomas, uncovering potential probe targets. Thus, molecular probes may provide a highly targeted labeling technique for the fluorescence visualization of glioma.

#### Peptide-Based Labels

BLZ-100 or tozuleristide (4,766 Da) is a protein composed of chlorotoxin (CTX), a 36-residue neurotoxic peptide found in scorpion venom, conjugated to ICG. As a neurotoxin, CTX works to block small-conductance chloride channels, triggering internalization by endocytosis (281). CTX has been shown to selectively localize to gliomas via inhibitory binding of matrix metalloproteinase 2 that is frequently upregulated by glioma cells to facilitate tissue invasion (282). Thus, BLZ-100 is a high-affinity, targeted fluorescent probe that has shown specificity for glioma cells and is capable of near-infrared fluorescence, making it viable for applications of fluorescence-guided glioma resection. BLZ-100 has seen success in preclinical studies for resection of glioma (120), head and neck carcinoma (121), and soft-tissue sarcoma (122). Liposomes targeted with CTX were also tested in mouse glioblastoma (283). BLZ-100 has also passed a Phase I clinical trial, showing no toxicity for doses up to 30 mg (123). When administered in doses of >9 mg, BLZ-100 fluorescence was detected *ex vivo* or *in vivo* in 4 of 7 grade-2 gliomas and 4 of 4 grade-4 gliomas, demonstrating potential transport across the relatively competent BBTB in these tumors and optimism for sensitive and specific brain tumor labeling. Currently, BLZ-100 is undergoing a joint Phase II/III trial for fluorescence-guided resection of pediatric CNS tumors (NCT03579602).

Another fluorescent molecular probe that falls in this category is IRDye 800CW-cyclic-RGD. This probe employs a recognition motif containing a tripeptide sequence (Arg-Gly-Asp) that binds integrin receptors that are overexpressed on the tumor surface. Huang et al. (124) tested this near-infrared probe in three mouse glioblastoma models, including a transgenic glioblastoma mouse model (RCAS-PDGF-driven/tv-a glioblastoma), which mimics the infiltrative growth pattern of human glioblastomas and associated heterogeneity of the BBTB. In this model, the label demonstrated great delineation of tumor margin and tumor cells.

#### Affibody-Based Labels

Elliott et al. (108) reported on the simultaneous administration of an anti-EGFR affibody conjugated to a near-infrared fluorescent probe, named ABY-029 (7,914.95 Da), a marker of perfusion, IRDye680RD (927.13 Da), and 5-ALA, for the fluorescent visualization of F98 orthotopic gliomas in rats. All three labels were administered simultaneously, 3 h before imaging, thus relying on passive targeting and EPR mechanisms. The results of the study showed significant but complementary differences in staining patterns of the three markers. As measured by histological analysis, ABY-029 performed expectedly better in the visualization of EGFR-expressing tumors (91% accuracy, 80% overall accuracy), while 5-ALA performed better in the visualization of the tumor margins of non-EGFR-expressing tumors (87% accuracy, 84% overall accuracy). ABY-029 is currently undergoing a Phase I clinical trial for the fluorescent-guided resection of recurrent glioma (NCT02901925). We expect this affibody to have a good correlation with a gadolinium-based contrast imaging based on similar molecular weights and therefore expected similarity in crossing the BBTB.

#### Antibody-Based Labels

Martirosyan et al. (109) reported the use of an FITC-conjugated anti-EGFR antibody (~70,000 Da) for the identification of EGFR-expressing F98 tumor cells using confocal laser endomicroscopy in rats 24 h after IV administration of the FITC-conjugated antibodies. Warram et al. (110) investigated IRDye 800CW conjugated to an anti-EGFR antibody for fluorescence guidance in orthotopic mouse gliomas 3 days after IV administration. In both studies, EGFR-expressing tumor cells were readily visualized *in vivo*, confirming the viability of antibody-conjugated fluorescent tumor identification in the presence of a BBTB. While these studies are preclinical, there is significant potential for the application of this technique clinically for resection of high-grade glioma, but not for low-grade glioma, where the BBTB more closely resembles an intact BBB. Future applications of this technique may be as a part of a theranostic approach, whereby characterization of tumor protein expression guides the selection of appropriate antibodies for targeted fluorescence visualization.

#### Liposomal-Based Labels

Liposomes represent a class of highly modifiable nano- to micro-scale carriers that have a bi-lipid membrane. The size, composition of the lipid membrane, surface charge, mechanical properties, and anchoring of biologically active ligands can each be adjusted to optimize pharmacokinetics (141). Importantly, some liposome formulations are already FDA approved.

Jia et al. (125) investigated ICG-laden, biomimetic proteolipid liposomes (BLIPO-ICG, 104 ± 3 nm size) for the fluorescence-guided resection of C6 orthotopic mouse gliomas. These nanoparticles were infused with cell-surface proteins harvested from C6 glioma cells, enabling them to evade phagocytosis and to precisely target tumor cells via the homotypic interaction of surface proteins. Further, the authors were able to achieve complete surgical resection of tumors, guided by BLIPO-ICG and validated with histology. These results did not extend to resection guided by control nanoparticles without tumor-derived cell-surface markers, indicating that the cell-surface proteins contributed to greater tumor-specificity. Despite this, the invasiveness of C6 glioma cells in this study likely does not accurately replicate the invasiveness of high-grade human gliomas. As such, further testing is needed to evaluate the effectiveness of this nanoparticle beyond intact regions of BBTB and for further clinical applicability.

The design of fluorescent molecular probes for brain tumor visualization warrants consideration of the wide variation in structural characteristics of these molecules as they relate to interactions with the BBB. The barrier properties of the CNS endothelium severely restrict passive diffusion of molecules across the BBB, and transporters such as the neonatal Fc receptor and ABC-type efflux pumps work to actively remove antibodies and other large molecules from the brain. Absent any mechanisms for selective targeting of probes to tumor cells, accumulation via the EPR effect is likely not sufficient to stain high-grade brain tumor margins and low-grade gliomas, which are the main areas of difficulty for current fluorescent markers. As such, methods to functionalize markers for delivery across the BBB are warranted to ensure adequate BBB permeability and marker accuracy.

### Functionalizing Particles for BBB Passage in Gliomas

Crucially, functionalization of nanoparticles can allow for effective extravasation despite an intact BBB, increasing the utility of fluorescence-guided glioma resection. Identifying a family of peptides with structural homology to the ligands that induce endothelial transcytosis is an important step to deliver drugs across the intact BBB and BBTB endothelium. ANG2, a member of the angiopep family of proteins, was identified to exhibit high transcytosis capacity via LRP-1 and is actively used for functionalization of various molecular vehicles that can carry therapeutic or diagnostic agents or both (112).

Hao et al. (113) demonstrated the efficacy of a combined chemo-phototherapy technique using ANG2-coated polylactide-co-glycolide (PLGA) nanoparticles loaded with ICG and the microtubule toxin docetaxel (DTX) (ANG2/PLGA/DTX/ICG probe) in a U87MG mouse orthotopic glioma model. Ni et al. (114) investigated an ANG2-targeted dual MRI-optical nanoprobe consisting of a fluorescent up-conversion nanoparticle loaded with gadolinium (ANG2/PEG-UCNP probe) in an orthotopic mouse glioblastoma model. The ANG2 coating facilitated the selective uptake of these labels across the BBB in both studies, as visualized by fluorescence imaging.

Ma et al. (115) described a fluorescent probe conjugated with TGN, a BBB targeting peptide selected from a library of phage display. In their experiments, TGN was conjugated with glioma targeting aptamer AS1411 and Cy3 orange fluorescent dye and showed some glioma cell-labeling capacity in an orthotopic C6 mouse glioma model.

These nanoparticles are examples of an extensively functionalized drug-delivery system, where a BBB-targeting motif potentially allows for better labeling of the marginal tumor zone. Unfortunately, all three studies were performed in orthotopic glioblastoma models that display disrupted BBTB, which might not fully represent the heterogeneous BBTB and especially its competent regions. The additional complexity of the probes raises further concerns about stability, toxicity, and clearance that have to be further investigated. Overall, functionalization of the labels with BBB-targeted molecules is a significant advance for trans-BBB drug delivery.

## Conclusion

The optimal surgical treatment of invasive brain tumors requires accurate visualization of tumor margins to maximize cytoreductive resection within functionally safe borders. Optically guided brain tumor surgery is an intriguing method for improving resection and has seen a number of exciting advancements within the last few years. Despite the initial enthusiasm in some of these techniques, the diversity and heterogeneity of gliomas complicate the consistent and accurate labeling of all tumors and limit clinical success. These difficulties require visualization agents to cross the BBB or BBTB to reach target tumor tissues. Not only do these barriers physically limit passive diffusion of many therapeutics, but also the dynamic expression of a vast network of transporters and junctional proteins further complicates drug delivery to gliomas. Thus, the efficacy of labels for optically guided glioma surgery will vary depending on tumor- and patient-specific BBB and BBTB properties. In this review, we described the barrier properties of the CNS and gliomas and discussed a number of technologies that have potential in overcoming these barriers for better fluorescence-visualization of gliomas.

Many novel tumor-targeted labels that have a large molecular weight do not cross competent BBTB regions in low-grade gliomas. Given that these areas of competent BBB or BBTB are where currently used markers struggle, it is important that novel tumor labels address this impediment. Novel BBB disruption techniques could be used to improve brain tumor labeling using these labels. Alternative BBB-targeted drugs with optical imaging properties are promising new tools. While many of these technologies are still in the preclinical stages of development and, therefore, require additional time and development before they may be available clinically, these advances offer a number of solutions for BBB-mediated brain tumor labeling and therapy.

## Author Contributions

EB and CL: conception and design. KS, EB, VB, and CL: acquisition of data and drafting the article. All authors critically revised the article. All authors reviewed and edited the manuscript. LC and MP: supervision. All authors approved the final version of the manuscript.

## Conflict of Interest

The authors declare that the research was conducted in the absence of any commercial or financial relationships that could be construed as a potential conflict of interest.
